# ModelCIF Update: Supporting Emerging Classes of Computational Macromolecular Models^☆^

**DOI:** 10.1016/j.jmb.2026.169658

**Published:** 2026-01-22

**Authors:** Gerardo Tauriello, Yoriko Lill, Jacopo Sgrignani, Vincent Zoete, Benedikt Singer, Brinda Vallat, Benjamin M. Webb, Thomas Garello, Stefan Bienert, Michael Feig, Elena Papaleo, Stephen K. Burley, Andrej Sali, Markus A. Lill, Andrea Cavalli, Matteo Dal Peraro, Torsten Schwede

**Affiliations:** 1 Biozentrum, University of Basel, Basel, Switzerland; 2 SIB Swiss Institute of Bioinformatics, Switzerland; 3 Department of Pharmaceutical Sciences, University of Basel, Basel, Switzerland; 4 Institute for Research in Biomedicine, Università della Svizzera italiana (USI), Faculty of Biomedical Sciences, Bellinzona, Switzerland; 5 Department of Oncology UNIL, Ludwig Institute for Cancer Research, University of Lausanne, Lausanne, Switzerland; 6 Institute of Bioengineering, School of Life Sciences, Ecole Polytechnique Fédérale de Lausanne (EPFL), Lausanne, Switzerland; 7 Research Collaboratory for Structural Bioinformatics Protein Data Bank, and the Institute for Quantitative Biomedicine, Rutgers, The State University of New Jersey, Piscataway, NJ 08854, USA; 8 Rutgers Cancer Institute, Rutgers, The State University of New Jersey, New Brunswick, NJ 08901, USA; 9 Department of Bioengineering and Therapeutic Sciences, the Quantitative Biosciences Institute (QBI), and the Department of Pharmaceutical Chemistry, University of California, San Francisco, San Francisco, CA 94157, USA; 10 Department of Biochemistry & Molecular Biology, Michigan State University, MI, USA; 11 Cancer Structural Biology, Danish Cancer Institute, Copenhagen, Denmark; 12 Cancer Systems Biology, Department of Health Technology Bioinformatics, Technical University of Denmark, Lyngby, Denmark; 13 Research Collaboratory for Structural Bioinformatics Protein Data Bank, San Diego Supercomputer Center, University of California, La Jolla, CA 92093, USA; 14 Department of Chemistry and Chemical Biology, Rutgers, The State University of New Jersey, Piscataway, NJ 08854, USA; 15 Rutgers Artificial Intelligence and Data Science (RAD) Collaboratory, Rutgers, The State University of New Jersey, Piscataway, NJ 08854, USA

**Keywords:** data standard, macromolecular structure prediction, protein complexes, conformational states, protein design

## Abstract

The recent development of highly accurate protein structure prediction tools has led to a rapid expansion in the scope of computational structural biology, enabling a much wider range of modelling studies than ever before. These new *in silico* opportunities help life science researchers understand how proteins interact with their environment and support design of new molecules with desired properties. Ultimately, they have broad applications, e.g. in medicine, drug discovery or engineering. To ensure reproducibility and to facilitate data exchange and reuse, predicted structures or computed structure models can be stored using ModelCIF, a rich data representation designed to include the atomic coordinates/metadata. The previously published version of ModelCIF (1.4.4; 2022–12-21) mainly covered protein structure predictions generated by homology and *ab initio* modelling. In this work, we present an extension of the ModelCIF (https://github.com/ihmwg/ModelCIF) data standard and its associated tools. This extension supports important new use cases, including modelling protein–ligand and protein–protein interactions, sampling multiple conformational states and designing proteins *de novo*. We define guidelines for storage and validation of modelling results for those use cases by applying new and existing ModelCIF categories to capture protocols, inputs and outputs. Additionally, we outline updates to the software tools and resources that implement these new standards and provide functionality for model generation, validation, archiving, and visualisation. By enabling consistent metadata capture across different modelling workflows, this framework aims to support the FAIR dissemination of computational models, thereby promoting reproducibility and reusability in downstream applications.

## Introduction

Atomic-level structural information on biological macromolecules is fundamental to our mechanistic understanding of the life sciences, as well as to a range of applications, including the study of molecular mechanisms, drug discovery, enzyme engineering and synthetic biology. In recent years, the quantity and quality of computed structure models (CSMs) has increased dramatically, transforming the way researchers generate and utilise structural hypotheses. Breakthroughs in deep learning-based protein structure prediction, most notably AlphaFold2 [1], have catalysed this shift by delivering monomer models that are often of near-experimental accuracy, and by generating a rich ecosystem of methods for end-to-end *in silico* studies that extend beyond single-chain fold prediction [2–7]. However, the results of these studies must be complemented by relevant metadata if they are to be shared and reused in a FAIR (findable, accessible, interoperable and reusable) manner.

This evolution of modelling methods has two immediate implications. Firstly, the range of models that can be produced accurately has broadened to include protein–ligand complexes, multi-macromolecular assemblies, models in different conformational states, and models for novel designed sequences and structures. Secondly, the methods used to produce these models are more varied, ranging from AI-powered co-folding and physics-based docking to investigations based on molecular mechanics and complex protein design pipelines. Consequently, the metadata required to enable interpretation, reproducibility, and reuse in subsequent processes now extends well beyond what is needed for 'classic' monomeric predictions.

ModelCIF [8] is an extension of PDBx/mmCIF designed specifically for the FAIR archiving and sharing of CSMs and their metadata. Building on interoperable PDBx/mmCIF coordinate representations, ModelCIF adds modelling-specific categories for protocols, inputs, quality assessment and provenance. Models in the ModelCIF format can be deposited in ModelArchive [9], a public, citable resource for deposition and dissemination that has experienced significant growth in submissions since the release of AlphaFold2 in 2021. Unlike the Protein Data Bank (PDB) [10] and PDB-IHM [11], which focus on archiving experimentally determined structures and integrative/hybrid models tied to experimental restraints, the scope of ModelArchive and ModelCIF is centred on computational outputs. It is also not intended for full molecular dynamics trajectories, nor for explicitly representing intrinsic disorder beyond what can be captured by static models in multiple states, since these cases are handled by other repositories [12–14]. Compared to other databases containing CSMs, such as the AlphaFold database [15], the SWISS-MODEL Repository [16], and other commercially maintained databases such as ProteinBase from Adaptyv Bio [17], this work expands the scope of data storage by enabling these databases to also employ the new ModelCIF format, which adheres to the FAIR principles.

**Figure 1.**
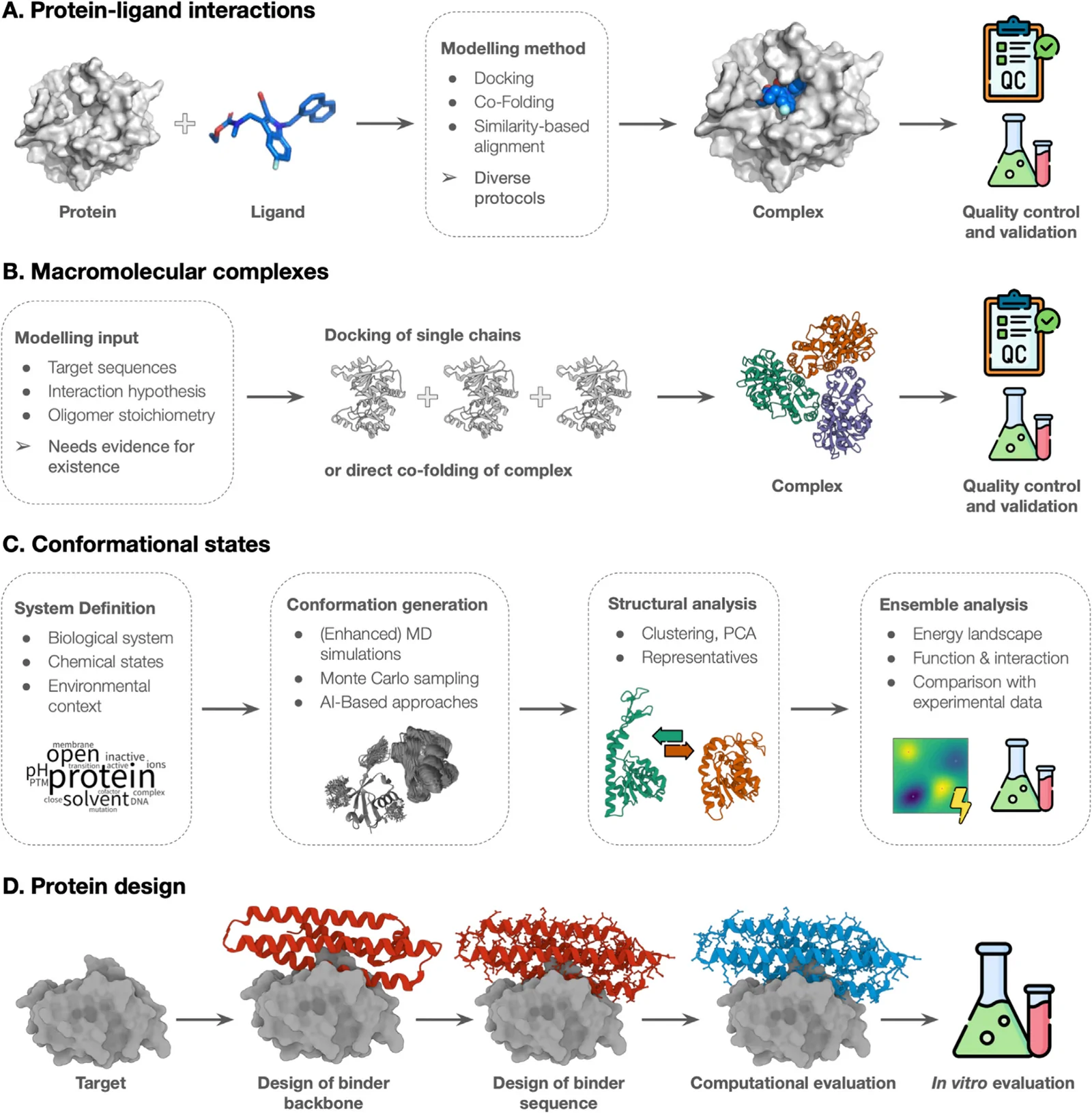
Example workflows for emerging modelling use cases supported by the extended ModelCIF standard: (A) Protein-ligand interactions: docking or co-folding, followed by pose evaluation; (B) Macromolecular complexes: assembly generation and interface assessment; (C) Conformational states: sampling alternative states, then selecting representatives; (D) Protein design: backbone and sequence design, followed by structural evaluation.

Despite the strong foundation provided by ModelCIF, the rapid evolution of computational structural biology has exposed gaps. In order to address these gaps, targeted extensions to the data standard are required, as well as precise guidance on how to use existing definitions to consistently capture machine-actionable metadata. To outline the challenges we face and our contributions, we present four representative use cases (Figure 1) that are becoming increasingly common.

### Protein-ligand interaction models

In these studies, a small molecule is modelled in complex with a macromolecular target. Compared to protein targets, ligands require a more explicit description of their chemical identity and state. This includes topology, atom and bond typing, protonation state and, in some workflows, the initial 3D conformation. The protocols employed are diverse and include docking, co-folding, and template-guided approaches [18–20]. This results in heterogeneous metadata requirements and quality assessments. Additionally, FAIR archiving must capture multiple complementary validation criteria and, where available, include links to supporting experimental evidence (e.g. binding affinity or complex formation measurements).

### Interactions between macromolecules

Modelling assemblies is central to understanding function, but this approach introduces two validation layers: (1) Do the partners interact, and if so, what is the stoichiometry? and (2) Are the predicted interfaces accurate? Historically, the predominant approach was to dock preformed subunits [21], whereas modern AI methods can co-fold partners directly to produce models of complexes [19,22–25]. Confidence metrics focusing on the relative orientation of molecules and contact geometry are required. These should be complemented by records of experimental evidence for the existence of the interactions.

### Modelling of distinct conformational states

Many macromolecular systems exist in multiple biologically relevant states (e.g. open/closed or active/inactive) that are frequently influenced by their environment. Computational protocols used to explore these states include AI-based sampling [26,27], homology modelling, and *ab initio* modelling [28], as well as physics-based exploration [29]. These protocols result in multiple states, which can be equally accurate and relevant, occupying regions along a conformational landscape. These models can be organised into groups and accompanied by energy estimates for the states.

### Protein design

Current protein design pipelines consist of the following *in silico* steps [6]: backbone generation [30–32]; inverse folding or sequence generation [33,34]; and validation, where protein structure prediction methods are mainly used to judge and rank the designs structurally [19,22,23]. Finally, *in vitro* testing is critical for evaluating the expression, stability, and functional features of these proteins. The pipeline can also be fully integrated with additional optimisation steps to increase the *in vitro* success rate [35–37].

### ModelCIF update

This work addresses the needs identified by the community by proposing targeted extensions to the ModelCIF data dictionary (https://github.com/ihmwg/ModelCIF) and providing guidelines driven by use cases for applying new and existing definitions consistently and interoperably to capture protocols, inputs and outputs. By anchoring these advances in ModelCIF, we aim to strengthen the FAIR ecosystem for computational structural biology. Specifically, our contributions facilitate the precise description of what was modelled (entities, states and conditions), how it was modelled (protocols, software, parameters and inputs) and why the results can be trusted and how they can be reused (fit-for-purpose quality assessments and links to experimental evidence). The rest of this article outlines the guidelines for storing and validating models for each use case, and summarises updates to the tools and resources that support the generation, validation, archiving, and visualisation of models. Together, these developments help to ensure that, as computational structural biology continues to expand, its outputs will remain accessible, interpretable, usable and durable for the wider research community.

## Results and Discussion

### Storing metadata in ModelCIF

ModelCIF builds on PDBx/mmCIF to represent computed structure models with sufficient context to enable FAIR reuse and reproducibility while remaining flexible enough to cover a rapidly expanding spectrum of modelling studies. The core of ModelCIF consists of one or more modelling protocol steps, described in free text and linking input and output data and the software used. In the following, we propose a series of practical guidelines for four use cases that extend beyond the scope of conventional protein structure predictions: protein–ligand interactions, macromolecular complexes, multiple conformational states, and protein design pipelines (Figure 1). Our recommendations and schema updates are based on input from expert practitioners and ModelArchive users across the four domains. We conducted surveys, interviews and focused workgroup discussions with experts, who provided real-world examples and advice. This process emphasised the value of focused additions of metadata that materially improve interpretability and reproducibility without placing an undue burden on model depositors.

With regard to protein–ligand interactions, it is essential that metadata include unambiguous chemical identity and explicit documentation of the small molecule utilised for modelling. Depositors are encouraged to utilise identifiers in accordance with the wwPDB chemical component dictionary [38] or to define the small molecule directly in the ModelCIF file by providing the canonical SMILES or InChI representation and IUPAC name of the ligand and by including references to external databases such as PubChem [39] or ChEMBL [40]. If a specific 3D conformation or protonation state has been employed in the modelling process, it is possible to provide an accompanying SDF or CIF file as part of the associated data. It is crucial that the protocol includes a comprehensive list of the software and versions utilised, in addition to the key software parameters that govern the modelling outcomes.

In macromolecular complexes, the modelled stoichiometry and interaction hypothesis must be made explicit in the metadata. Existing ModelCIF constructs can be used to encode classical docking, molecular dynamics and AI-based co-folding methods, provided the metadata include the modelled molecular entities and well-described protocol steps, as well as a list of software and their key parameters. Free text descriptions of protocol steps can be used to provide additional information on the stoichiometry and interaction hypothesis. If possible, this should be complemented by experimental evidence showing that the entities are expected to interact, and this evidence can be stored in a new ModelCIF category, as described in the 'Validation' section.

Many systems are best described by multiple conformational states (e.g. open/closed or active/inactive). PDBx/mmCIF natively supports multi-model entries, which are commonly used for NMR ensembles. ModelCIF now builds on this by enabling users to optionally group models into annotated clusters, with specific models defined as cluster representatives according to selection criteria such as lowest energy within the group or proximity to the cluster centroid. Protocol details should capture decisions that impact the sampled state space, such as the protonation states of titratable residues, the pH value, the ionic strength of the environment and the sampling methodology used. If these details differ between model groups, this can be made explicit in the cluster annotations.

Finally, protein design pipelines present a number of unique challenges. Design campaigns usually involve the following steps: (i) generation of the backbone, (ii) design of the sequence/inverse folding and (iii) validation/triage of the structure, often with iterative refinement. ModelCIF can already represent these steps using the protocol and data categories, without the need for design-specific file types. Archiving every intermediate backbone and candidate sequence can be excessive and of limited reuse value, so we discourage it. Instead, depositors should focus on the final selected model(s) and: (a) describe the end-to-end pipeline, including the software used, its versions, and the key parameters; (b) provide an overview of the generation scales (e.g. the number of backbones and sequences produced); (c) indicate the selection filters used to narrow down the set (e.g. structure confidence, foldability, and interface metrics); (d) store the coordinates for the final selected model(s).

### Validation criteria for extended use cases

**Figure 2.**
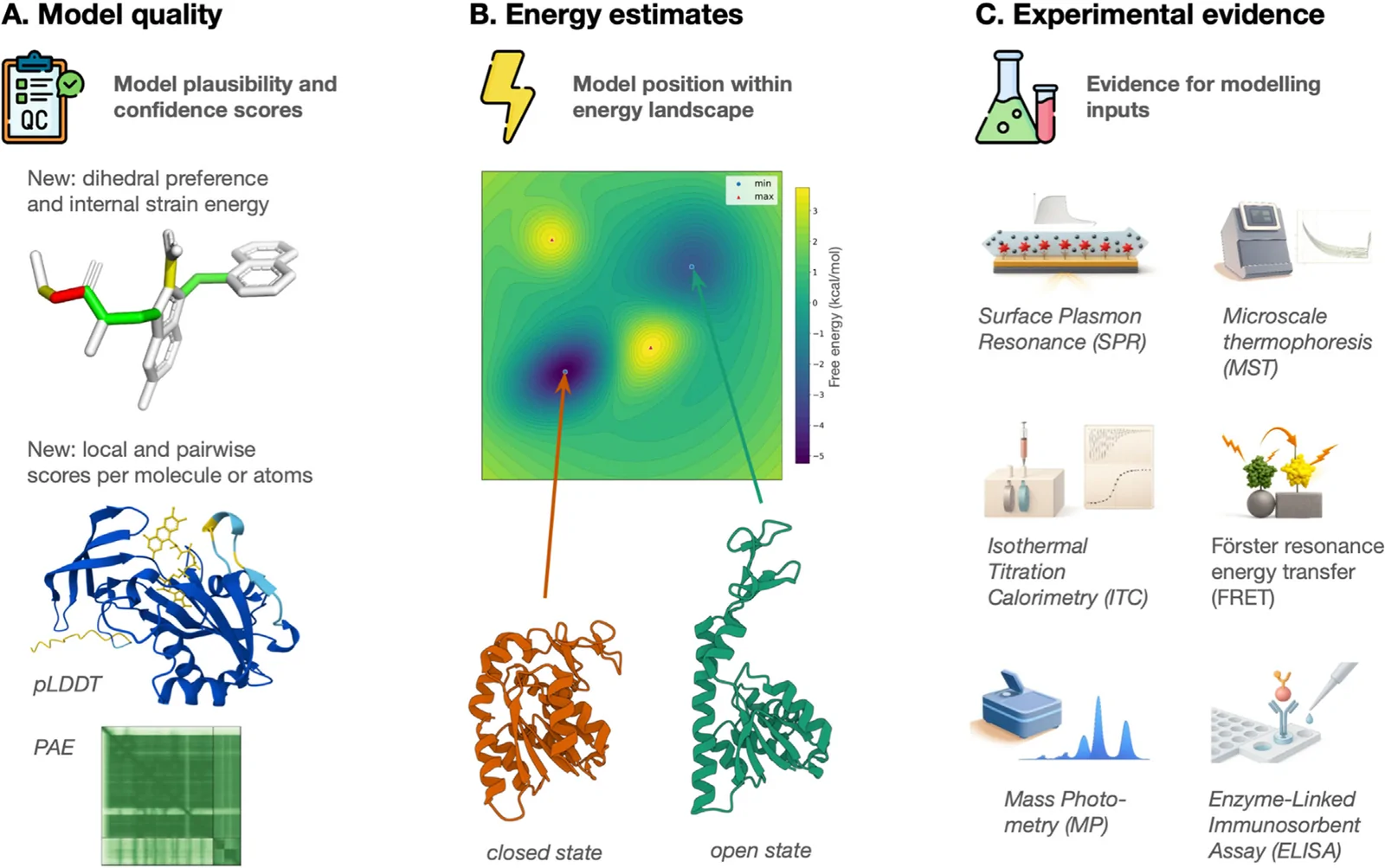
Summary of validation aspects supported by the extended ModelCIF standard. These include new features to capture (A) model plausibility and confidence scores, (B) energy estimates and (C) experimental evidence for modelling inputs.

Validation is essential for building trust in computational models and enabling their informed selection for subsequent use. ModelCIF now supports the storage of validation data that goes beyond global and per-residue estimates of model quality (or prediction confidence). Validation data can be computed by modelling software or via independent methods and linked to protocol steps to make provenance explicit. Figure 2 provides an overview of the newly added validation criteria.

The advent of all-atom co-folding methods, such as AlphaFold3 [19], Boltz-1 [23], Chai-1 [24], and Protenix [25], meant that the granularity of quality estimate scores had to be increased. ModelCIF now enables scores to be defined for individual features and feature pairs. Each feature can represent one or more atoms, polymer residues, or instances of a molecular entity. This supports all confidence metrics used by recent co-folding methods, including pairwise scores such as PAE, which are defined per token pair and where a token may represent either a single atom or a polymer residue.

While evidence for the existence of most molecular entities can be found by cross-referencing external knowledge bases such as UniProtKB [41] or ChEMBL [40], the existence of a specific molecular complex comprising a certain number of instances of each molecular entity, or the ability to fold a designed protein sequence, cannot be assumed. To address this issue, ModelCIF now supports the storage of experimental evidence for the existence of molecular entities and the complexes formed from them. We also allow for negative evidence in studies where models were generated to evaluate the ability of methods to distinguish between interacting and non-interacting molecules. The experimental evidence supported in ModelCIF is distinct from the experimental data that leads to the restraints used for integrative modelling, the impact of which is immediate on the modelling itself. Experimental data for restraints are handled by IHMCIF [42] for deposition in the PDB-IHM branch of the PDB archive.

Validation of protein–ligand interactions covers three areas: (1) confidence in the protein model (e.g. pLDDT and PAE) at the binding site; (2) plausibility of the ligand conformation, including torsion preferences and internal strain; and (3) quality of the interaction at the protein–ligand interface. To support the results of tools such as TorsionAnalyzer [43], which assess torsional quality per dihedral angle, ModelCIF now allows quality estimate scores to be defined per dihedral angle. Information on specific interactions between protein and ligand atoms (e.g. hydrogen bonds and steric clashes), as well as estimates of internal strain energy and per-ligand-atom confidence, can be stored using ModelCIF quality estimate scores. These can be stored globally, per-residue, per-feature or in pairs. As there is no single, universally accepted non-experimental quality measure for validating protein–ligand models, ModelCIF encourages the addition of multiple quality assessment criteria and experimental evidence of the interaction's existence and binding affinity.

For macromolecular complexes obtained by co-folding methods, global and local confidence indicators such as pLDDT, pTM and ipTM are commonly used. However, they can be influenced by non-interacting or disordered regions. Metrics focused on the interface, such as ipSAE [44], actifpTM [45] or pDockQ2 [46], better capture the relative positioning of subunits and the plausibility of the binding interface. These should be reported either globally or locally for each pair of molecules. Wherever possible, we recommend storing experimental evidence of macromolecular interactions and the stoichiometry of the complex.

When modelling multiple conformational states accurately, it is not possible to categorise them as 'better' or 'worse'. Rather, they occupy distinct regions of the energy landscape, some possibly corresponding to biological states. In addition to the model quality estimates described previously, ModelCIF introduces new categories that allow different energies to be stored for each conformation. These include conformational free energies, interaction energies and solvation energies, among others.

Protein design pipelines can use model quality estimates to select designs from large sets of candidates, and the relevant scores can be stored in ModelCIF. In order to evaluate these proteins generated *in silico*, it is crucial to perform high-throughput expression and purification, followed by *in vitro* validation [47,48]. Experimental evidence from the final validation steps can be stored in ModelCIF and may include assessments of whether the expressed design is properly folded and monomeric, as well as measurements of binding affinity and catalytic efficiency. Some of these models eventually undergo structural determination, in which case they can be linked to PDB entries.

### Supporting software tools and resources

The evolution of ModelCIF is accompanied by a growing ecosystem of standards, software libraries, validators, and archival services that together operationalise the guidelines above.

ModelCIF is a community developed standard, closely aligned with PDBx/mmCIF. Updates are curated by the ModelCIF working group and made available via the public dictionaries at https://github.com/ihmwg/ModelCIF and https://mmcif.wwpdb.org/dictionaries/mmcif_ma.dic/Index/. The dictionary contains annotated definitions of all ModelCIF categories and data items, and the GitHub repository includes detailed documentation with examples of how to use ModelCIF in practice. Recent versions incorporate the new categories mentioned above, such as model grouping and representatives, quality estimates at feature level and for dihedrals, experimental validation and energy estimates. They also propagate enhancements from the core PDBx/mmCIF dictionary, ensuring compatibility with PDBx/mmCIF files that use recent changes. The python-modelcif library provides programmatic authoring of ModelCIF files and includes convenience classes to simplify this process. A collection of ModelCIF conversion scripts based mostly on python-modelcif, as well as a ModelCIF validation tool based on RCSB’s CifCheck, are available at https://git.scicore.unibas.ch/schwede/modelcif-converters. Visualisation tools such as Mol* and ChimeraX support ModelCIF and can display PAE matrices or per-residue confidence values extracted from ModelCIF, among other things.

The ModelArchive (https://modelarchive.org/) is a repository for computational structure models that are not based on experimental data and is powered by ModelCIF. It continues to evolve in order to accommodate the above guidelines, offering flexible deposition workflows and the FAIR dissemination of models. If the necessary metadata is available, existing models in ModelCIF format can be remediated to support ModelCIF's new capabilities. The ModelArchive already contains sets of models with protein–ligand complexes (e.g. ma-tbvar3d), macromolecular complexes (e.g. ma-idk-u2osmap) and protein design (e.g. ma-osf-ppp2r2a). These were made possible by free text descriptions in ModelCIF, but future sets for these use cases will include metadata as described in this work. Thanks to ModelCIF's alignment with PDBx/mmCIF, interoperability with structures based on experimental data (PDB and PDB-IHM) is preserved, and infrastructures such as 3D-Beacons [49] can be used to distribute models to external services.

## Conclusion and Perspectives

The field of computational structural biology continues to advance rapidly with no signs of slowing down. ModelCIF data standards need to be continuously revisited to capture relevant metadata for newly developed methods and approaches used by the scientific community. Looking forward, we envision tighter interoperation with PDB-IHM for integrative modelling scenarios and expanded support for model sets. The latter could be based on PDBe’s InvestigationCIF dictionary which was developed for fragment screening investigations but could be adapted to capture multiple models generated for the same study. ModelArchive contains a number of model sets of this type and additionally we observe studies (e.g. virtual screening) where a large number of models are produced but only a subset of high quality models are stored. A description of the full investigation could then capture both the stored models and also the discarded ones with information of the modelling setup and the model confidence scores used to filter them.

Community feedback from expert users, collected via surveys and hands-on data curation in ModelArchive, will continue to guide pragmatic extensions that keep the standard simple where possible and specific where necessary. The tools and resources surrounding ModelCIF are evolving to simplify the adoption of the format and the archiving of models with relevant metadata. We aim to add functionality to programmatically convert quality estimate files from tools like AlphaFold into ModelCIF, to provide support for uploading multiple ligand poses in SDF format and converting them to ModelCIF, to implement automated consistency checks between molecular entities and cross-references, and to include validation results from stereochemistry checks and tools like TorsionAnalyzer into ModelCIF.

By pairing a stable core with targeted, use-case-driven additions, the ModelCIF ecosystem is well positioned to support reproducible, reusable computational models in a rapidly evolving field.

## Data Availability

No data was used for the research described in the article.
